# Mosquito cell line glycoproteins: an unsuitable model system for the *Plasmodium *ookinete-mosquito midgut interaction?

**DOI:** 10.1186/1756-3305-3-22

**Published:** 2010-03-25

**Authors:** Simon Wilkins, Peter F Billingsley

**Affiliations:** 1 Department of Biology, Imperial College of Science Technology and Medicine, South Kensington, London, SW7 2BB, UK; 2 Monash Institute of Medical Research, Monash University, Melbourne, VIC, 3168, Australia; 3 Sanaria Inc., 9800 Medical Center Drive, Suite A209, Rockville, MD, 20850, USA

## Abstract

**Background:**

Mosquito midgut glycoproteins may act as key recognition sites for the invading malarial ookinete. Effective transmission blocking strategies require the identification of novel target molecules. We have partially characterised the surface glycoproteins of two cell lines from two mosquito species; *Anopheles stephensi *and *Anopheles gambiae*, and investigated the binding of *Plasmodium berghei *ookinetes to carbohydrate ligands on the cells. Cell line extracts were run on SDS-PAGE gels and carbohydrate moieties determined by blotting against a range of biotinylated lectins. In addition, specific glycosidases were used to cleave the oligosaccharides.

**Results:**

*An. stephensi *43 and *An. gambiae *55 cell line glycoproteins expressed oligosaccharides containing oligomannose and hybrid oligosaccharides, with and without α1-6 core fucosylation; N-linked oligosaccharides with terminal Galβ1-3GalNAc or GalNAcβ1-3Gal; *O*-linked α/βGalNAc. *An. stephensi *43 cell line glycoproteins also expressed *N*-linked Galβ1-4R and *O*-linked Galβ1-3GalNAc. Although *P. berghei *ookinetes bound to both mosquito cell lines, binding could not be inhibited by GlcNAc, GalNAc or Galactose.

**Conclusions:**

Anopheline cell lines displayed a limited range of oligosaccharides. Differences between the glycosylation patterns of the cell lines and mosquito midgut epithelial cells could be a factor why ookinetes did not bind in a carbohydrate inhibitable manner. Anopheline cell lines are not suitable as a potential model system for carbohydrate-mediated adhesion of *Plasmodium *ookinetes.

## Background

Malaria is caused by parasitic protozoa of the genus *Plasmodium *which are transmitted to humans by *Anopheles spp*. (Diptera:Culicidae) mosquitoes. The life cycle of *Plasmodium *is complex with asexual development in the human host as well as sexual and asexual development in the mosquito [1]. Strategies for the development of malaria vaccine candidates target the stages found within humans e.g. the asexual erythrocytic stages, however vaccine candidates have also been developed against both the sexual stages of malaria in the mosquito (reviewed in [2]) and against the mosquito vector itself (reviewed in [3]). The most developed candidate vaccine is RTS, S which can reduce the multiplicity of infection in African adults and is currently the subject of a Phase 3 trial initiated in May 2009 [4]. In the mosquito, the main barrier to the ookinete is the mosquito midgut and passage through the midgut epithelium is the critical step for successful establishment of a *Plasmodium *infection in the mosquito [5,6]. Successful transmission is dependent on vector:parasite compatibility [7], as well as mosquito age and parasite density in the blood meal [8].

Oligosaccharides on midgut glycoproteins are receptors for microbial attachment in a number of insects systems [9-11]. Mosquito midgut glycoproteins typically are heavily glycosylated with a high proportion of *N*-linked GlcNAc- and GalNAc-terminal oligosaccharides [12] and carbohydrates are key elements in the interaction of both *Plasmodium falciparum *and *Plasmodium vivax *in *Anopheles tessellatus *[13], and *Plasmodium gallinaceum *in *Aedes aegypti *[14]. More recent work has identified a family of lectin-like proteins termed *Plasmodium berghei *lectin adhesive-like protein (PbLAP1-5) expressed in *P. berghei *ookinetes and gametocytes which could be involved in binding carbohydrate ligands on the midgut epithelial cell surface [15]. Sugar epitopes may provide potential transmission blocking targets as a monoclonal antibody raised against a GalNAc containing midgut glycoprotein blocked *Plasmodium yoelli *infectivity in *Anopheles stephensi *[16]. Further identification of effective transmission blocking strategies requires a fuller understanding of the molecular interactions between the parasite and the mosquito cells, and this includes the exposed carbohydrate structures [17].

Cell lines have been used as model systems for lectin-mediated adhesion of parasites, for example *Entamoeba histolytica *binding to Caco-2 cells and *Giardia lamblia *binding to Int-407 cells [18,19]. The cell lines were substitutes for the usual host cells and the interactions between the parasites and the cell lines were inhibited by carbohydrates. In an insect system, the identification of a midgut carbohydrate ligand for *Bacillus thuringiensis *toxin [20], has led to studies using insect cell lines in binding assays to understand the molecular interaction between the *B. thuringiensis *toxin and the host cell [21].

In the present study, mosquito cell lines were investigated as a potential model system for *Plasmodium *ookinete binding to the mosquito midgut. Initially the glycosylation profile of the membrane glycoproteins of two anopheline mosquito cell lines was investigated. Secondly we assessed whether ookinetes would bind to the cell lines in a carbohydrate-dependent manner. Identification of any ookinete binding ligand molecules would be a useful step in the progress towards identification of vaccine target molecules to prevent the transmission of malaria by mosquitoes.

Using an array of lectins and glycosidases we show the presence of a number of different oligosaccharides on glycoproteins from two anopheline cell lines; *An. stephensi 43 *and *An. gambiae 55*. In addition we show that *P. berghei *ookinetes bind to monolayers of both anopheline cell lines but the binding is not inhibitable by monosaccharides. Finally we discuss the implications of the different pattern of glycosylation observed in the cells compared with the mosquito midgut, the ookinete binding to the cell lines, and the suitability of the cell lines as a model for the ookinete:mosquito midgut interaction.

## Methods

### Mosquito cell lines

Two mosquito cell lines, *An. stephensi *43, and *An. gambiae *55, were derived from embryonic larval cells [22,23]. Cell lines were cultured at in an incubator at 28°C in medium optimised for cell growth. Cell lines were tested 20-26 passages from liquid nitrogen stocks (*An. stephensi *43 cell lines -stock passage number 210; *An. gambiae *55 cell lines -stock passage number 150). Both *An. stephensi *43 and *An. gambiae *55 cell lines were cultured in a 1:1 mix of Modified Kitamura's/Varma and Pudney's VP_12 _medium (MKVP_12_) [22] supplemented with 10% (v/v) heat inactivated foetal calf serum (Gibco) and .0.5% (v/v) penicillin-streptomycin-neomycin solution (Gibco).

### Sample preparation

A T75 culture flask (Gibco) for each cell line was grown to near confluence. Cells were dislodged from the flask surface with a cell scraper (Gibco) and the cell suspension added to a 15 ml Falcon tube and spun at 800 rpm to pellet the cells. The supernatant was discarded and cell pellet was resuspended and centrifuged twice at 800 rpm with physiologically buffered saline (PBS; 0.15 M NaCl, 0.027 M KCl, 0.01 M Na_2_HPO_4_, pH 7.4) to remove cell culture media. Cells were homogenised in a 0.1 ml glass/glass homogeniser (Jencons) in PBS containing 5% v/v Triton X-100 (Sigma). Protein concentrations were calculated against a Bovine Serum albumin (BSA Fraction V; Sigma) standard [24].

### SDS-PAGE and lectin blotting

Mosquito cell line samples containing 2 μg protein in 10 μl PBS TX-100 were loaded with an equal volume of sample buffer onto 10 or 12.5% SDS-PAGE gels [25]. Proteins were transferred onto 0.45 μ Nitrocellulose membrane (NCM) and stained in 0.1% w/v Ponceau S in 5% v/v acetic acid (Sigma). Marker lanes were removed and stained with colloidal gold (Protogold^®^, British BioCell International) according to manufacturer's instructions. NCMs were blocked overnight at 4°C in TBS-T (0.1% v/v 0.05 M Tris-HCl, 0.15 M NaCl, 0.1 v/v % Tween 20, pH 7.5). Transferred lanes containing mosquito and control glycoproteins were cut lengthways into strips for multiple testing of a single lane. NCMs were incubated for 45 min in 20 μg/ml biotinylated lectin solution (all lectins sourced from Vector Labs, UK), rinsed, and then incubated for 45 min in streptavidin-linked alkaline phosphatase (0.2 U/ml) in TBS-T. Alkaline phosphatase (AP; Vector Labs., UK) was developed according to manufacturers instructions using a combination of BCIP (5-Bromo-4-Chloro-3'-Indolyl Phosphate sodium salt) and NBT (Nitro-Blue Tetrazolium chloride) as a substrate (Vector Labs., UK). Lectin binding controls consisted of lectin only or streptavidin-linked AP only. Positive control glycoproteins were used as controls for both lectin blotting and sugar inhibitions. These were prepared by either running glycoproteins (2 μg per well) on SDS-PAGE gels and transferring to NCM as described above or glycoproteins (5-10 μg) were dot-blotted directly onto NCM then blocked before use. Molecular weights (kDa) of mosquito cell line proteins were estimated using protein marker standards from 3-5 different experiments. Lectins used in this study, their respective positive controls, and specificities are shown in Table 1. Abbreviations for lectins used in this study: AAL, *Aleutia aurantia *agglutinin; Con A, Concanavalin A; DBA, *Dolichos biflorus *agglutinin; JAC, *Artocarpus integrifolia *agglutinin; MAL_II_, *Maackia amurensis *lectin; PHA, *Phaesolus vulgaris *agglutinin; RCA_II_, *Ricinus communis *agglutinin; PNA, Peanut agglutinin; SBA, Soybean agglutinin; SNA, *Sambuca nigra *agglutinin; UEA_I_, *Ulex europaeus *agglutinin; WGA, wheat germ agglutinin.

**Table 1:** Lectins used and their specificities

Lectin	Abbreviation	Sugar specificity	Positive control	Reference
**Concanavalin A**	Con A	α-mannose, α-glucose	SBA, Ovalbumin	[46]
**Wheat Germ Agglutinin**	WGA	(GlcNAc)_2_, Sialic acid, GalNAc	Ovalbumin	[47]
***Dolichos biflorus *agglutinin**	DBA	α-GalNAc	ASM	[48]
**Soybean agglutinin**	SBA	α, β-GalNAc, Gal	ASM	[49]
**Peanut agglutinin**	PNA	Galβ1-3GalNAc	ASM, BSA-T	[50]
***Artocarpus integrifola *agglutinin**	JAC	Galβ1-3GalNAc, GalNAcβ1-3 Gal, GalNAc	ASM, BSA-T	[51,52]
***Phaesolus vulgaris *agglutinin**	PHA	Galβ1-4GlcNAc	ASF	[53]
***Ricinus communis *agglutinin**	RCA_II_	Galβ1-4GlcNAc	ASF	[54]
***Maackia amirensis *lectin**	MAL_II_	Sialic acidα2-3Gal	Mouse blood	[55]
***Sambuca nigra *agglutinin**	SNA	Sialic acidα2-6Gal	Fetuin	[56]
***Ulex europaeus *agglutinin**	UEA_I_	Fucα1-2Gal	Mucin	[57]
***Aleutia aurantia *agglutinin**	AAL	Fucα1-3/6	Mucin	[58]

Positive controls for each lectin were dot blotted and treated as described in materials and methods.

### Carbohydrate inhibition

Cell samples and control glycoproteins were prepared and transferred onto NCM as described above. Lectin staining of mosquito cell line glycoproteins and controls was carried out in the presence of mono- and di- saccharides to inhibit lectins and thereby confirm the specificity of the interactions between the lectins used and the oligosaccharides. Each sugar was added to biotinylated lectin solutions at an excess concentration of 100 mM to ensure complete abrogation of lectin binding. Lectin staining was carried out as described above.

### Glycosidase treatments

Cell samples and control glycoproteins were prepared and transferred onto NCM as described above. Digestion of exposed oligosaccharides *in situ *was carried out using an Immunetics miniblotter (Cambridge, MA, USA) to deliver 125 μl of reaction mixture containing endo- or exo-glycosidases directly onto the NCM strips; optimum enzyme concentrations and specificities are shown in Table 2. Enzymes were added in excess amounts to ensure full cleavage during treatments. The blotter was sealed and incubated for 24 h at 37°C in a humid atmosphere under constant gentle agitation. After extensive rinsing with TBS, strips were stained with biotinylated lectins as described above.

**Table 2:** Summary of glycosidases and running conditions used for digestion of mosquito cell line glycoproteins.

Enzyme (enzyme number)	Abbreviation	Cleavage site	Enzyme Conc.	Incubation Buffer (pH)	Reference
**Peptide-*N*-glycosidase F** **(EC 3.2.2.18)**	PNGase F	*N*-linked oligosaccharides-Asn	50 U	0.5 M sodium phosphate (7.5)	[59]
**Peptide-*N*-glycosidase A** **(EC 3.5.1.52)**	PNGase A	*N*-linked oligosaccharides-Asn	2.5 mU	50 mM sodium citrate, sodium phosphate (5.0)	[60]
**Endo-β-glucosaminidase H** **(EC 3.2.1.96)**	Endo H	Oligomannose/ hybrid oligosaccharides-Asn	50 U	0.5 M sodium citrate (5.5)	[61]
**Endo-α-acetylgalactosaminidase** **(EC 3.2.1.92/3.2.1.110)**	O-glycanase™	Galβ1-3GalNAcα1-Ser/Thr	82-112 mU	10 mM sodium citrate, sodium phosphate (6.0)	[62]
**α-*N*-acetylgalactosaminidase** **(EC 3.2.1.49)**	α-AGA	GalNAcα1-3R/Ser/Thr	625 mU	100 mM sodium citrate, sodium phosphate (3.5-4)	[63]
**β-Galactosidase** **(EC 3.2.1.23)**	-	Galβ1-3GlcNAc -Glc/Man	4 mU	20 mM sodium citrate, sodium phosphate (6.0)	[64]
**Neuraminidase** **(EC 3.2.1.18)**	-	Sialic acidα2-3,6,8R	25 mU	0.5 M sodium citrate (4.5)	[65]

### Chemical deglycosylation of MV glycoproteins

NCMs containing transferred cell line glycoproteins were cut lengthways into two strips, rinsed in 50 mM sodium acetate buffer (pH 4.5) and then treated with 20 mM periodic acid in buffer for 1 h in the dark at 23°C. NCMs were rinsed with acetate buffer then incubated in 50 mM sodium borohydride in PBS for 30 min at room temperature. Strips were then washed in TBS-T and lectin stained.

### *Plasmodium berghei *ookinete cultures

*P. berghei *(clone 2.34L) (originally from Dr. D. Walliker, University of Edinburgh) were maintained by cyclical passage in female Theiler's original (TO) mice and *An. stephensi *mosquitoes, and ookinetes prepared as described previously [26]. Parasitaemia levels were monitored by Giemsa stained blood smears and slides were examined under a light microscope ×40 objective using oil immersion.

### Ookinete: mosquito cell line binding assays

MTT (3- [4,5-dimethylthiazol-2-yl)-2,5-diphenyl tetrazolium bromide) is a yellow substrate which is cleaved by active mitochondria in living cells to yield a purple end product, therefore dead cells are not detectable using this substrate [27]. Initially a standard curve of MTT production against ookinete number was calculated. Ookinete numbers were estimated on a haemocytometer. Ookinetes were then diluted in PBS and a two-fold serial manner, and MTT (Sigma; 5 mg/ml sterile filtered in PBS) was added (10 μl per 100 μl medium/PBS) to each dilution, and the mixtures vortexed and incubated for 4 h at 37°C. Acid-isopropanol (100 μl of 0.04 N HCl in isopropanol (BDH) or an equal amount of the initial volume) was added to each well to dissolve MTT crystals. Cell debris was pelleted and the supernatant plated out in triplicate onto 96 well plates (Nunc). Plates were read on either an Anthos HTIII or Molecular Devices Vmax microtitreplate reader at 570 nm and at the reference wavelength of 620 nm. Absorbance values at 620 nm were subtracted from the absorbance values at 570 nm.

Binding assays were set up using anopheline mosquito cell lines. Numbers of cultured cells were estimated on a haemocytometer and 300,000 cells were added to form a monolayer in sterile 96 well plates (Greiner). Excess medium was then removed and the plates washed with PBS using a Vacu-pette 96 (DBM Scientific) and the cells fixed with methanol for 30 s. Non-specific protein binding sites were then blocked with 1% w/v BSA in PBS for 1 h at 37°C, and the cells were further washed three times with PBS as above. Ookinete cell numbers per μl of culture were estimated on a haemocytometer and 20,000 ookinetes were added per well and incubated on cell monolayers for 6 h at 19°C. A 6 h incubation time was chosen to match the escape time of mature ookinetes from the mosquito midgut lumen [28]. Following incubation, plates were washed three times in PBS as above, then 100 μl PBS were added to each well. MTT assay was carried out as described above with controls of a PBS blank and fixed cells alone. Binding assays were carried out three times with 5-6 technical replicates for each data point. Data were analysed by one way ANOVA with post hoc Dunnett's test in GraphPad Prism.

The toxicity of carbohydrates to be used in binding assays were tested in Eppendorf tubes by adding 500 μl of monosaccharide solutions to 500 μl ookinete cultures containing approx. approximately 20,000 ookinetes. Carbohydrates were tested at concentrations of 100 mM, 250 mM and 500 mM. Ookinetes were exposed to the carbohydrates for 6 h at 19°C then an MTT assay carried out as above.

## Results

### Proteins of mosquito cell line samples

**Figure 1:**
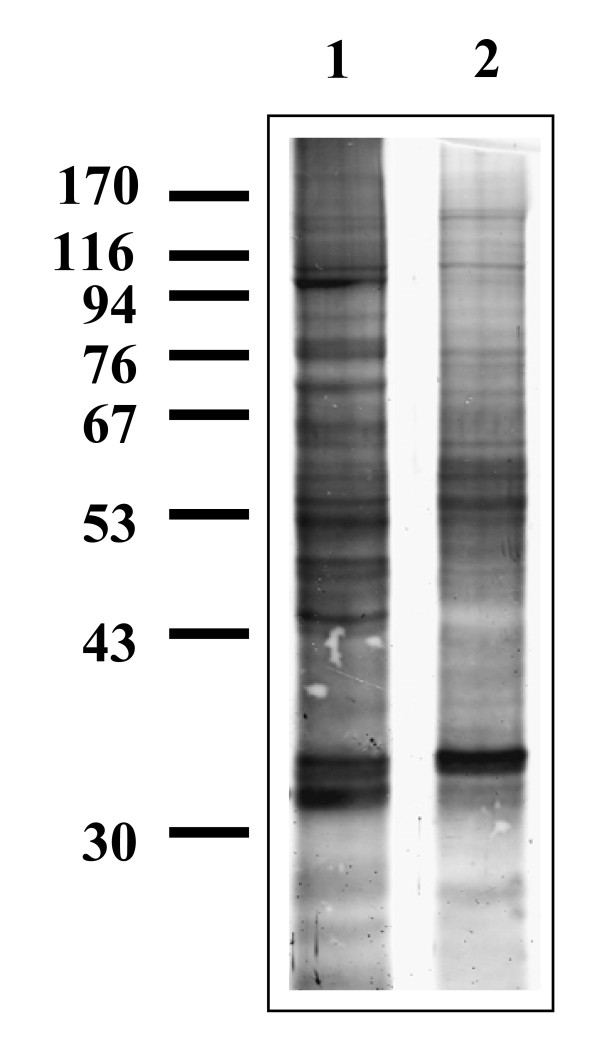
**Gold staining of mosquito cell line protein samples**. Lane 1 *An. stephensi *43 cell line protein sample (4 μg loaded); lane 2 *An. gambiae *55 cell line protein sample (4 μg loaded). Molecular weight marker positions are shown in kDa. Representative blot shown of 4 separate experiments.

Anopheline cell line extracts contained proteins that were detected with Protogold^® ^(Figure 1), but did not bind any lectins tested. The *An. stephensi *43 cell line sample contained twelve major bands detected only with Protogold^® ^(approximately 206, 133, 103, 92, 77, 68, 63, 53, 50, 46, 28, and 27 kDa)(Figure 1, lane 1) and an additional twenty six which were detected with lectins. Ten major bands were detected with Protogold^® ^in the *An. gambiae *55 cell line sample (approximately 145, 108, 77, 74, 58, 55, 53, 35, 34, and 30 kDa)(Figure 1, lane 2) in addition to sixteen proteins detected with lectins.

### Characteristics of *An. stephensi *43 cell line glycoproteins

**Figure 2:**
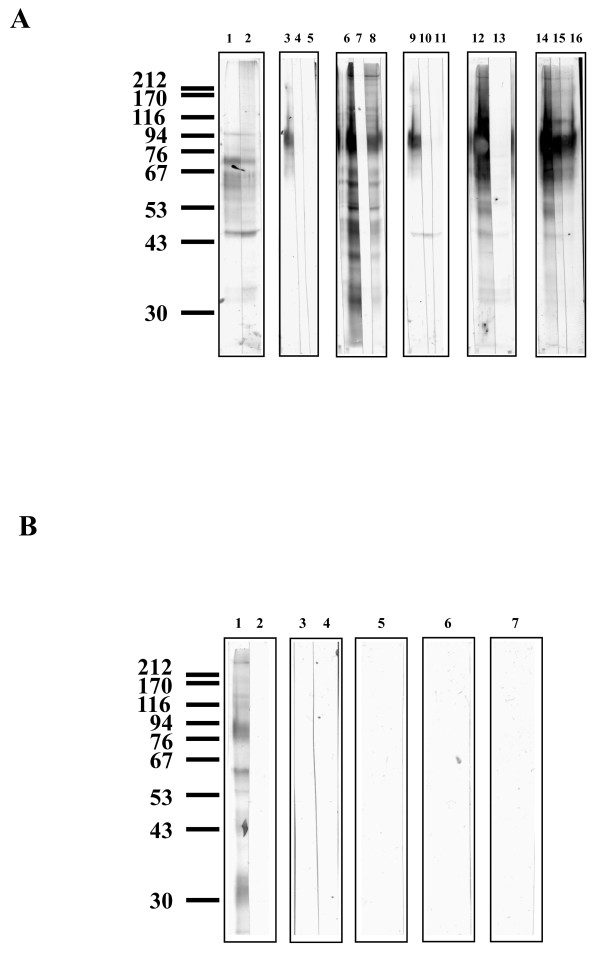
**Lectin blotting with a range of lectins to *An. stephensi *43 cell line glycoproteins with and without competitive sugar inhibitions**. The position of molecular weight markers run concurrently are shown, values in kDa. Two μg of protein were loaded to each well. **Figure 2A: **Lane 1 SNA, and with 100 mM Sialic acid (lane 2); lane 3 RCA_II_, with 100 mM galactose (lane 4), with 100 mM melibiose (lane 5); lane 6 Con A, with 100 mM mannose (lane 7), with 100 mM glucose (lane 8); lane 9 PNA, with 100 mM galactose (lane 10), 100 mM melibiose (lane 11); lane 12 JAC, with 100 mm GalNAc (lane 13); lane 14 JAC, with 100 mM galactose (lane 15), 100 mM melibiose (lane 16). **Figure 2B: **Lane 1 AAL, with 100 fucose (lane 2); lane 3 WGA; with 100 mM GlcNAc (lane 4), lane 5 UEA_I _; lane 6 MAL_II _; lane 7 PHA. Representative blot shown of 3-5 separate experiments.

**Figure 3:**
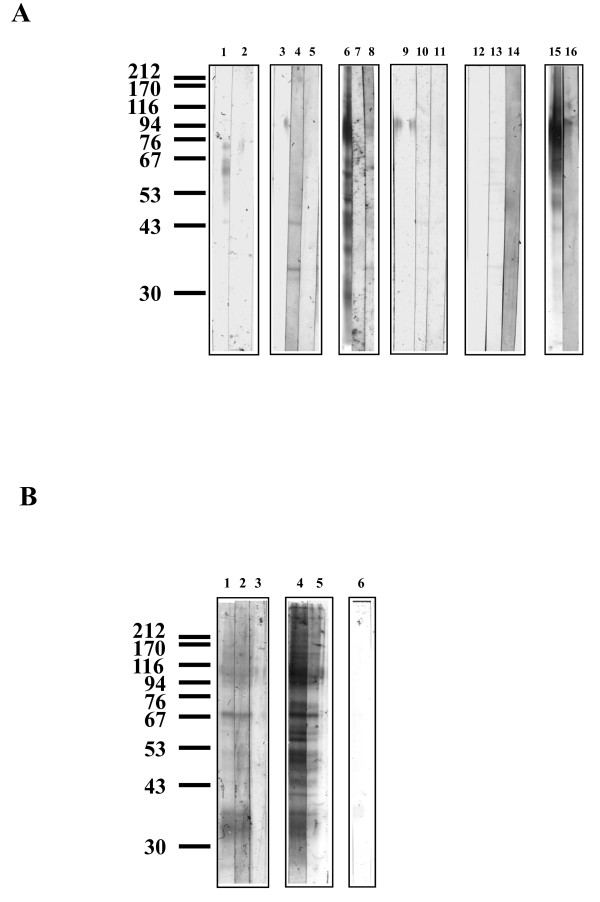
**Lectin blotting with a range of lectins to *An. stephensi *43 cell line glycoproteins before and after *in situ *glycosidase treatments**. The position of molecular weight markers run concurrently are shown, values in kDa. Two μg of protein were loaded to each well. **Figure 3A: **Lane 1 SNA, after neuraminidase (lane 2); lane 3 RCA_II_, after β-galactosidase (lane 4), after PNGase F (lane 5); lane 6 Con A, after PNGase F (lane 7), after Endo H (lane 8); lane 9 PNA, after *O*-glycanase™ (lane 10), after PNGase F (lane 11); lane 12 DBA, after α AGA (lane 13), after PNGase F (lane 14); lane 15 JAC, after PNGase F (lane 16). **Figure 3B: **Lane 1 AAL, after Endo H (lane 2), after PNGase A (lane 3); lane 4 Con A, after PNGase A (lane 5), after periodate (lane 6). Representative blot shown of 3-5 separate experiments.

Six lectins bound to glycoproteins from *An. stephensi *43 cells; Con A, SNA, RCA_II_, PNA, JAC and AAL (Figure 2A and 2B). WGA, UEA_I_, MAL_II _and PHA did not bind to any glycoprotein (Figure 2B). Additionally SBA did not recognise any glycoprotein (data not shown). Twelve major bands (approximately 370, 138, 117, 103, 87, 67, 59, 52, 48, 45, 39, and 33 kDa) and two minor bands (approximately 33.5 and 30 kDa) were recognised by Con A (Figure 2A, lane 6). Binding to each of these glycoproteins was abolished by 100 mM mannose and reduced by 100 mM glucose (Figure 2A, lanes 7 and 8). *In situ *digestion with PNGase F removed all Con A binding (Figure 3A, lane 7). Endo H treatment removed Con A binding, with the exception of the 87, 59, and 33 kDa glycoproteins, where binding was only partially reduced (Figure 3A, lane 8). PNGase A reduced Con A binding intensities of all glycoproteins (Figure 3B, lane 5), but to a lesser extent than Endo H. Chemical deglycosylation with periodate removed all Con A binding (Figure 3B, lane 6). AAL bound strongly to six glycoproteins from *An. stephensi *43 cells (approximately. 370, 138, 117, 87, 79, and 59 kDa) and also faintly to four glycoproteins (approximately. 52, 45, 42, and 36 kDa) (Figure 2B, lane 1). Competitive inhibition by 100 mM fucose removed AAL binding to all glycoproteins (Figure 2B, lane 2). *In situ *treatment with PNGase F removed all AAL binding sites from the glycoproteins (data not shown) whereas *in situ *Endo H and PNGase A did not alter binding of AAL (Figure 3B, lanes 2 and 3).

JAC bound to nine glycoproteins, four that were strongly stained (approximately 370, 122, 111, and 87) and five that were only faintly stained (approximately 55, 52, 48, 34 and 33) (Figure 2A, lane 12 and 14). Binding to the 370, 122, and 87 kDa glycoproteins was abolished in the presence of 100 mM GalNAc (Figure 2A, lane 13), but only reduced in the presence of 100 mM galactose and 100 mM melibiose (Figure 2A, lanes 15 and 16). Binding to the 55 and 52 kDa glycoproteins was inhibited by 100 mM GalNAc and 100 mM melibiose and partially inhibited by 100 mM galactose. The binding intensity of the 48 kDa glycoprotein was reduced with 100 mM GalNAc and 100 mM galactose but abolished with 100 mM melibiose. The 34 and 33 kDa glycoproteins had reduced binding intensities with 100 mM GalNAc but both 100 mM galactose and 100 mM melibiose inhibited binding (Figure 2A, lanes 13, 15 and 16). *In situ *treatment with *O*-glycanase™ did not alter binding intensity (data not shown), whereas PNGase F reduced the binding intensity of JAC to all glycoproteins (Figure 3A, lane 15 and 16). DBA did not bind to any glycoprotein and treatments *in situ *with α AGA and PNGase F revealed several faintly staining bands (Figure 3A, lanes 12, 13 and 14).

Both RCA_II _and PNA bound to an 87 kDa glycoprotein, and binding was inhibited competitively by 100 mM galactose and 100 mM melibiose (Figure 2A). Treatment with PNGase F or β-galactosidase removed binding of RCA_II _to the glycoprotein. However, two new bands were detectable at 44 and 33 kDa after β-galactosidase treatment (Figure 3A, lane 4). Treatments with *O*-glycanase™ and PNGase F removed PNA and RCA_II _binding to the 87 kDa glycoprotein (Figure 3A, lanes 10 and 5 respectively). The sialic acid-specific lectin SNA, bound to four glycoproteins (approximately 95, 72.5, 61,5 and 44 kDa) and two which were only faintly stained (approx. 60 and 58 kDa) (Figure 2A, lane 1). Competitive sugar inhibition with sialic acid did not reduce binding, and an unexpected additional band was detectable at 33 kDa (Figure 2A, lane 2). *In situ *neuraminidase treatment abolished SNA binding sites (Figure 3A, lanes 1 and 2).

### Characteristics of *An. gambiae *55 cell line glycoproteins

**Figure 4:**
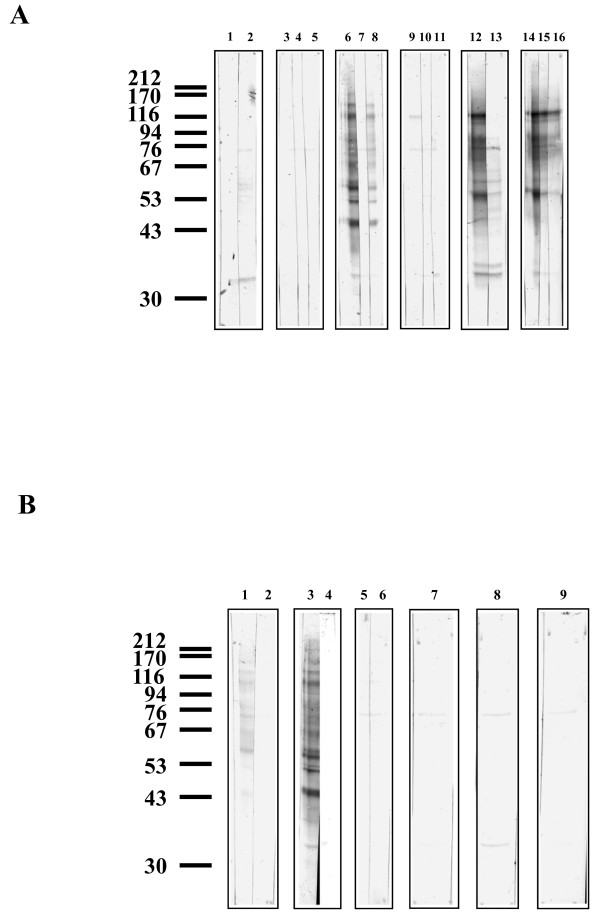
**Lectin blotting with a range of lectins to *An. gambiae *55 cell line glycoproteins with and without competitive sugar inhibitions**. The position of molecular weight markers run concurrently are shown, values in kDa. Two μg of protein were loaded to each well. **Figure 4A: **Lane 1 SNA, and with 100 mM Sialic acid (lane 2); lane 3 RCA_II_, with 100 mM galactose (lane 4), with 100 mM melibiose (lane 5); lane 6 Con A, with 100 mM mannose (lane 7), with 100 mM glucose (lane 8); lane 9 SBA, with 100 mM galactose (lane 10), 100 mM GalNAc (lane 11); lane 12 JAC, with 100 mM GalNAc (lane 13); lane 14 JAC, with 100 mM galactose (lane 15), 100 mM melibiose (lane 16). **Figure 4B: **Lane 1 AAL, with 100 fucose (lane 2); lane 3 Con A, and after periodate treatment (lane 4); lane 5 WGA, with 100 mM GlcNAc (lane 6); lane 7 UEA_I_; lane 8 MAL_II _; lane 9 PHA. Representative blot shown of 3-5 separate experiments.

**Figure 5:**
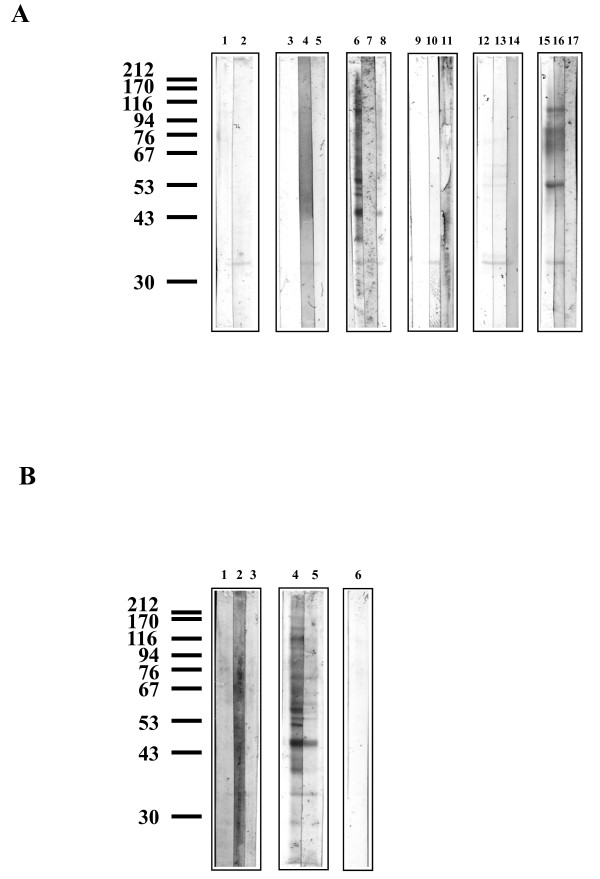
**Lectin blotting with a range of lectins to *An. gambiae *55 cell line glycoproteins before and after *in situ *glycosidase treatments**. The position of molecular weight markers run concurrently are shown, values in kDa. Two μg of protein were loaded to each well. **Figure 5A: **Lane 1 SNA, after neuraminidase (lane 2); lane 3 RCA_II_, after β-galactosidase (lane 4), after PNGase F (lane 5); lane 6 Con A, after PNGase F (lane 7), after Endo H (lane 8); after SBA (lane 9); after *O*-glycanase™ (lane 10), after PNGase F (lane 11); lane 12 DBA, after α AGA (lane 13), after PNGase F (lane 14); lane 15 JAC, after *O*-glycanase™ (lane 16), after PNGase F (lane 17). **Figure 5B: **Lane 1 AAL, after Endo H (lane 2), after PNGase A (lane 3); lane 4 Con A, after PNGase A (lane 5); lane 6 after periodate treatment. Representative blot shown of 3-5 separate experiments.

A different pattern of binding was observed with glycoproteins from *An. gambiae *55 cells, the lectins Con A, JAC, and AAL bound to several glycoproteins, and the lectins DBA, SBA, SNA, MAL_II_, RCA_II_, PHA, WGA, and UEA_I _faintly stained several glycoproteins (Figure 4A and 4B). Con A bound strongly to twelve glycoproteins (approximately 182, 143, 114, 101, 76, 65, 57.5, 55, 50, 48, 38, and 35 kDa), all of which except the 76 kDa band were no longer detectable after inhibition by 100 mM mannose (Figure 4A, lanes 6 and 7). Competitive inhibition by 100 mM glucose reduced Con A binding intensities to all glycoproteins (Figure 4A, lane 8). Both PNGase F and periodate treatments removed Con A binding to all glycoproteins (Figures 5A, lane 7 and Figure 4B, lane 4). Endo H treatment *in situ *reduced binding intensities of almost all glycoproteins except the 45 and 35 kDa glycoproteins which showed partial resistance to Endo H treatment (Figures 5A, lane 8). PNGase A treatment reduced the binding intensities of all glycoproteins (Figure 5B, lane 5).

MAL_II _and PHA bound very faintly to the 76 and 35 kDa glycoproteins (Figure 4B, lanes 8 and 9). SNA also bound to the 35 kDa glycoproteins (Figure 4A, lane 1), but in the presence of 100 mM sialic acid, the binding intensity of the 35 kDa glycoprotein had unexpectedly increased. Two additional bands (58 and 53 kDa) were detectable in the presence of 100 mM sialic acid (Figure 4A, lane 2). The 35 kDa glycoprotein was resistant (Figure 5A, lane 2). RCA_II _also bound to the 76 and 35 kDa glycoproteins, but 100 mM galactose and 100 mM melibiose did not inhibit binding (Figure 4A, lanes 3, 4, and 5). After β-galactosidase treatment a new band was detected with a molecular weight of 35 kDa (Figure 5A, lane 4). WGA bound very faintly to the 76 kDa glycoprotein, although this was not inhibited by 100 mM GlcNAc (Figure 4B, lane 5 and 6). AAL bound to four glycoproteins (approximately kDa 143, 114, 76, and 57.5 kDa), and binding was inhibited by 100 mM fucose (Figure 4B, lanes 1 and 2) and removed by PNGase F (data not shown). The fucose specific-lectin, UEA_I_, bound very faintly to the 35 kDa glycoprotein (Figure 4B, lane 7).

SBA bound faintly to three glycoproteins with molecular weights of 114, 76, and 35 kDa (Figure 4A, lane 9). Binding was not inhibited by either 100 mM galactose or 100 mM GalNAc (Figure 4A, lanes 10 and 11). DBA bound to glycoproteins with molecular weights of 76, 53.5, 36, and 35 kDa. DBA binding to these glycoproteins was not altered by either α AGA or PNGase F treatments, and the binding was very faint (Figure 5A, lanes 13 and 14). JAC bound strongly to several glycoproteins (approximately 114, 86.5, 76, 61, 57.5, 53.5, and 35 kDa) and faintly to the 101 kDa glycoprotein (Figure 4A, lane 12). Binding to the 114, 101, 76, and 57.5 kDa glycoproteins was inhibited by 100 mM GalNAc, whereas binding to the 61, 53.5, and 35 kDa glycoproteins was reduced by 100 mM GalNAc (Figure 4A, lane 13). Galactose inhibited binding to the 53.5 and 35 kDa glycoproteins, whereas 100 mM melibiose reduced the binding intensity of the 53.5 kDa glycoprotein and inhibited the binding of JAC to the 35 kDa glycoprotein. Binding to all other glycoproteins by JAC was not altered by 100 mM galactose and 100 mM melibiose (Figure 4A, lanes 15 and 16). *O*-glycanase™ did not reduce binding intensities, but *in situ *treatment with PNGase F removed all JAC binding (Figure 5A, lanes 16 and 17).

### Ookinete binding to anopheline mosquito cell lines

**Figure 6:**
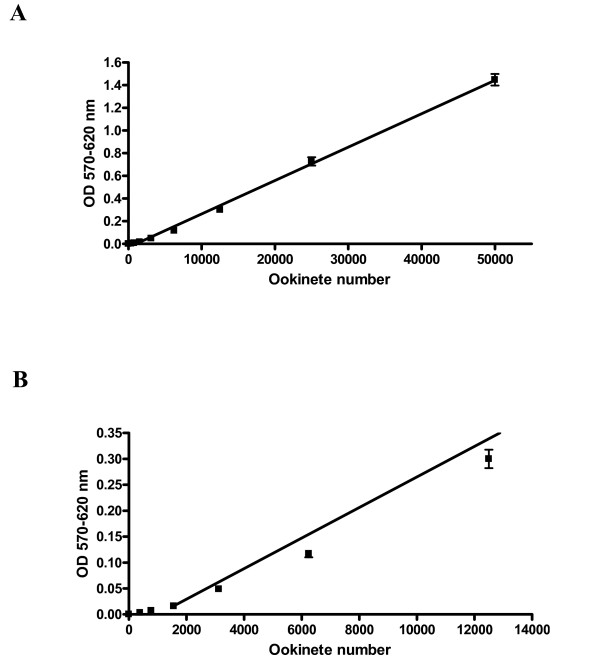
**Standard curve showing MTT cleavage against number of ookinetes**. Ookinete numbers were estimated on a haemocytometer. Ookinetes were then diluted in PBS and a two-fold serial manner, and MTT was(5 mg/ml) added to each dilution, and the mixtures vortexed and incubated for 4 h at 37°C. After incubation the resultant solutions were plated out on microwell plates. **Figure 6A: **Linear regression trend line is shown. Each point corresponds to the mean of six wells, bars indicate standard deviation of means. Trend line R^2 ^= 0.9974. **Figure 6B: **The lower limit of the linear range of the standard curve was at an absorbance reading of 0.02 which corresponds to around 1500 ookinetes.

**Figure 7:**
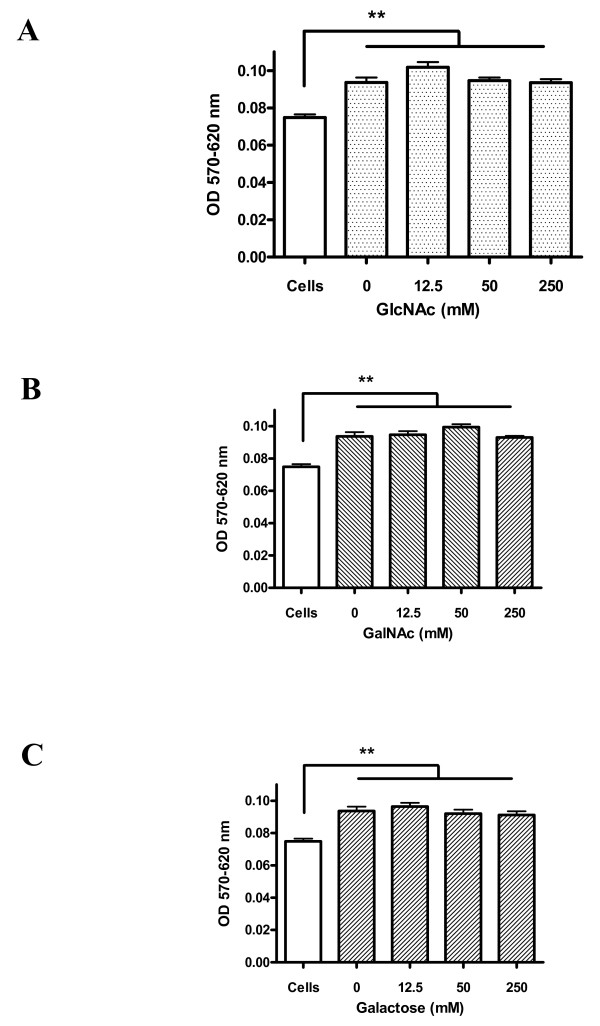
**Effect of different carbohydrates on the binding of *P. berghei *ookinetes to methanol-fixed *An. stephensi *43 cells**. Numbers of cells per ml of culture were estimated using a haemocytometer. 300,000 cells were added to each microwell, allowed to settle, then fixed with a 30 s treatment with methanol. 20,000 ookinetes in culture were added to each well and incubated in the presence or absence of carbohydrates for 6 h at 19°C. Following a washing step, 100 μl of PBS and 10 μl of MTT was added and the plates incubated for a further 4 h at 37°C. Plates were then read at 570 and 620 nm. Data shown are representative of three assays carried out. Plate readings were zeroed to a PBS blank. Shading indicates wells where ookinetes were added. ** Ookinete binding to cells was significantly higher than the control of cells alone (p < 0.01; One-way ANOVA, Dunnett's post hoc test). Data +/- SEM (n = 5-6). **Figure 7A: **Binding to cells alone, and GlcNAc at 0, 12.5, 50 and 250 mM. **Figure 7B: **Binding to cells alone, and GalNAc at 0, 12.5, 50 and 250 mM. **Figure 7C: **Binding to cells alone, and Galactose at 0, 12.5, 50 and 250 mM.

MTT production was linearly and directly proportional to the number of ookinetes seeded into microplate wells (Figure 6A). MTT production by ookinetes was not significantly reduced by monosaccharides at concentrations up to 500 mM (data not shown). A significant increase in MTT absorbance was detected when ookinetes were incubated with mosquito cells compared with MTT absorbance of mosquito cells alone (one-way ANOVA with post hoc Dunnett's test, p < 0.01) (Figure 7). However the change in absorbance values was small suggesting very weak binding and the absorbance values were at the limit of detection. Absorbance values (570nm-620 nm) of ~0.03 corresponded to binding of around 2000 ookinetes from the original 20,000 added per well (Figure 6B). Ookinete binding to fixed *An. stephensi *43 cells was tested in the presence of 12.5 mM, 50 mM, 250 mM GlcNAc, galactose and GalNAc. GlcNAc, galactose and GalNAc did not significantly reduce ookinete binding at any concentration tested (Figure 7). Binding to *An. gambiae *55 cells showed an identical profile of weak binding which was not significantly inhibited by carbohydrates (data not shown).

## Discussion

The use of plant-derived lectins to analyse and identify carbohydrates is a widely used technique employed in many different systems of host-parasite interactions [29,30]. Lectins have been essential and reliable tools in the investigation into malaria: mosquito interactions over a considerable period of time [31,32]. The aim of this study was to investigate the glycosylation of the membrane glycoproteins of two mosquito cell lines, with the intention of testing the cell lines as a potential model system for the carbohydrate-mediated interaction between the ookinete and mosquito midgut. Lectin binding coupled with respective sugar inhibitions, and glycosidase enzyme *in situ *treatments can provide much information about oligosaccharide structures present on glycoproteins from a given cell or tissue type [12].

The anopheline cell lines showed a limited glycosylation profile with only a few different types of oligosaccharides. A summary of oligosaccharides detected on *An. stephensi *43 cell line and *An. gambiae *55 cell line glycoproteins are shown in Tables 3 and 4 respectively. The most common, the oligomannose (Man_8-5_) was susceptible to both PNGase F and Endo H treatment and was present on fifteen *An. stephensi *43 cell line proteins, and twelve *An. gambiae *55 cell line proteins. Oligosaccharides were also present that were susceptible to PNGase F, Endo H and also bound AAL, a fucose specific lectin. The binding indicated the presence of core fucosylation when AAL binding was abolished with PNGase F treatment. However PNGase F is not capable of cleaving *N*-linked oligosaccharides that have α1-3 core fucosylation [33], so the fucosylation present on these glycoproteins may be α1-6 linked. This type of fucosylation is more typical of mammalian glycoproteins, although lepidopteran arylphorin and *Drosophila melanogaster *chaoptin glycoproteins express the same fucosylation linkage [34,35]. Chemical treatment with periodate before blotting removed all Con A binding sites on all mosquito cell line glycoproteins confirming the presence of adjacent hydroxyl groups within pyranose ring structures such as those found in mannose [36].

**Table 3:** Summary of oligosaccharides found on *An. stephensi *43 cell line glycoproteins

Glycoprotein approx. kDa	*N*-linked		*O*-linked
		
	Oligomannose/Hybrid	Complex	
370	Oligomannose/hybrid ± α1-6Fuc	Galβ1-3GalNAc GalNAcβ1-3Gal	GalNAcα/β-
138	Oligomannose/hybrid ± α1-6Fuc		
122		Galβ1-3GalNAc GalNAcβ1-3Gal	GalNAcα/β-
117	Oligomannose/hybrid ± α1-6Fuc		
111		Galβ1-3GalNAc GalNAcβ1-3Gal	GalNAcα/β-
103	Oligomannose		
87	Oligomannose/hybrid ± α1-6Fuc	Galβ1-3GalNAc GalNAcβ1-3Gal Galβ1-4R	Galβ1-3GalNAc GalNAcα/β-
79	Hybrid ± α1-6Fuc		
67	Oligomannose		
59	Oligomannose/hybrid ± α1-6Fuc		
55		Galβ1-3GalNAc GalNAcβ1-3Gal	GalNAcα/β-
52	Oligomannose/hybrid ± α1-6Fuc	Galβ1-3GalNAc GalNAcβ1-3Gal	GalNAcα/β-
48	Oligomannose	Galβ1-3GalNAc GalNAcβ1-3Gal	GalNAcα/β-
45	Oligomannose/hybrid ± α1-6Fuc		
42	Oligomannose/hybrid ± α1-6Fuc		
39	Oligomannose		
36	Oligomannose/hybrid ± α1-6Fuc		
34		Galβ1-3GalNAc GalNAcβ1-3Gal	GalNAcα/β-
33	Oligomannose		
33	Oligomannose	Galβ1-3GalNAc GalNAcβ1-3Gal	GalNAcα/β-
30	Oligomannose		

**Table 4:** Summary of oligosaccharides found on *An. gambiae *55 cell line glycoproteins

Glycoprotein approx. kDa	*N*-linked		*O*-linked
		
	Oligomannose/Hybrid	Complex	
182	Oligomannose		
143	Oligomannose/hybrid ± α1-6Fuc		
114	Oligomannose/hybrid ± α1-6Fuc	GalNAcβ1-3Gal	
101	Oligomannose/hybrid	GalNAcβ1-3Gal	
87		Galβ1-3GalNAc GalNAcβ1-3Gal	
76	Oligomannose/hybrid ± α1-6Fuc	GalNAcβ1-3Gal	GalNAcα/β-
65	Oligomannose		
61		Galβ1-3GalNAc GalNAcβ1-3Gal	
58	Oligomannose/hybrid ± α1-6Fuc	GalNAcβ1-3Gal	
55	Oligomannose		
54		Galβ1-3GalNAc GalNAcβ1-3Gal	GalNAcα/β-
50	Oligomannose		
45	Oligomannose		
38	Oligomannose		
36			GalNAcα/β-
35	Oligomannose	Galβ1-3GalNAc GalNAcβ1-3Gal	GalNAcα/β-

In both cell lines PNGase F reduced JAC binding, most clearly in *An. gambiae *55 cell line glycoproteins. This suggests that oligosaccharides were present with either GalNAcβ1-3Gal or Galβ1-3GalNAc residues were *N*-linked to the protein. Similar oligosaccharide structures have been detected previously in the venom of the honey bee *Apis mellifera *[37]. PNGase F reduced JAC binding to *An. stephensi *43 cell line glycoproteins suggesting some residues recognised by JAC were *O*-linked e.g. GalNAc linked directly to the protein. Binding of PNA was abolished by *O*-glycanase suggesting that Galβ1-3GalNAc was linked directly to the protein on the *An. stephensi 43 *cell line 87 kDa glycoprotein. Binding of DBA to *An. gambiae *55 cell line proteins suggested the presence of GalNAc residues *O*-linked directly to the protein.

Treatments with PNGase F and β-galactosidase prior to RCA_II _lectin binding indicated the presence of N-linked complex or hybrid oligosaccharides with terminal Galβ1-4GlcNAc on an *An. stephensi 43 *cell line glycoprotein. Although SNA bound to glycoproteins from both cell lines, sialic acid did not inhibit binding, and in some cases 100 mM Sialic acid appeared to enhance SNA binding to certain bands. It is not possible to draw firm conclusions as to the presence of sialic acid within the two mosquito cell lines. The presence or absence of sialic acid within insect cells has been a source of contention for many years (reviewed in [38]).

Oligosaccharides synthesised by insect cells differ from those synthesised by mammalian cells, for example an *Aedes albopictus *cell line, which lacks the transferases necessary to make complex-type oligosaccharides, add oligomannose structures to a protein glycosylation site which in mammalian cells would have a complex-type oligosaccharide added [39]. The anopheline cell lines used in this study showed a limited range of glycosylation consistent with other insect cell lines e.g. *Ae. aegypti *20A cells [40]. The fibroblast-like anopheline cells were derived from unknown tissues of first-stage mosquito larvae [22,23], and were originally cultured to be suitable for the study of malaria parasites and arboviruses [23]. The presence on mosquito cell line surfaces of a glycoprotein recognised by malarial ookinetes would provide a source of substrates for further molecular investigations of ookinete binding, a potential transmission blocking vaccine target, and a useful model for studying ookinete:mosquito midgut interactions *in vitro*. Identification of mosquito midgut surface carbohydrate epitopes have proved useful in understanding the interactions between the mosquito midgut and both arboviruses and the malaria parasite [16,41].

*P. berghei *ookinetes bound to both cell lines but although significant, the binding was at the limit of detection and at the lower limit of the linear relationship between MTT absorbance and ookinete number. Binding was not carbohydrate inhibitable with GlcNAc, GalNAc or galactose concentrations of up to 250 mM, suggesting a non-carbohydrate mediated binding. This binding could be through a ligand other than the oligosaccharides expressed on the mosquito cell line glycoproteins such as laminin or the proteoglycan chondroitin sulphate [42,43].

The question remains as to why anopheline cell lines did not provide a suitable model for ookinete binding. Although both cell lines expressed several oligosaccharides in common with the mosquito midgut, e.g. oligomannose, the primary and most important difference from the glycosylation of the mosquito midgut was the apparent lack of abundant terminal GlcNAc and GalNAc residues on cell line oligosaccharides. The interaction between the malarial ookinete and the mosquito midgut is complex and multi-faceted. Previous work suggested that there might be a lectin-like molecule on the ookinete surface [17]. Ookinetes of *P. berghei *bound to neoglycoproteins rich in GlcNAc residues, and GlcNAc and GalNAc reduced the infectivity of *P. berghei *ookinetes to *An. stephensi *mosquitoes [44,45]. The surface of *Ae. aegypti *and *An. stephensi *midgut microvilli are rich in both GlcNAc and GalNAc [12,31]. Carbohydrates could not be detected on either *P. gallinaceum *or *P. berghei *ookinetes [31]. This and other evidence suggests that a lectin-carbohydrate interaction exists and the lectin is on the ookinete surface [13,14,16]. It is likely that the lack of GlcNAc- and GalNAc-containing oligosaccharides from anopheline cell line glycoproteins was a contributing factor to the unsuitability of the cell lines as a model for ookinete:mosquito midgut binding.

In conclusion, although anopheline mosquito cell lines express some oligosaccharides common to the mosquito midgut, the cells are not a model system suitable for studying the binding of *P. berghei *ookinetes. These observations reinforce indirectly the importance of the presence of GlcNAc and GalNAc residues in the critical life cycle interaction between malarial ookinetes and the mosquito midgut.

## Conclusions

Using an array of lectins and glycosidases, we have shown the presence of a number of different oligosaccharides on glycoproteins expressed by two anopheline cell lines, *An. stephensi 43 *and *An. gambiae 55*. In addition we showed that *P. berghei *ookinetes bound poorly to monolayers of both anopheline cell lines but the binding was not inhibitable by monosaccharides. The different pattern of glycosylation observed in the cells compared with the mosquito midgut is a likely reason the ookinetes did not bind suggesting anopheline cell lines are not suitable as a model for the ookinete:mosquito midgut interaction. This study highlights the importance of GlcNAc and GalNAc oligosaccharides present on the mosquito midgut in the critical life cycle step for the malarial ookinete of traversing the midgut.

## Abbreviations

αAGA: α-*N*-acetylgalactosaminidase; AAL: *Aleutia aurantia *agglutinin; AP: alkaline phosphatase; ASF: asialofetuin; ASM: asialomucin; Asn: asparagine; BCIP: 5-bromo-4-chloro-3'-indolyl phosphate sodium salt; BSA-T: Galβ1-3GalNAc-Bovine serum albumin; Con A: Concanavalin A; DBA: *Dolichos biflorus *agglutinin; Endo H: endo-β-*N*-acetylglucosaminidase H; Fuc: fucose; Gal: galactose; GalNAc: *N*-acetylgalactosamine; GlcNAc: *N*-acetylglucosamine; Glc: glucose; JAC: *Artocarpus integrifolia *agglutinin; kDa: kilo Dalton; LPG: lipophosphoglycan (from *Leishmania major *promastigotes); M: melibiose; MAL_II_: *Maackia amurensis *lectin; Man: mannose; MTT: 3-[4,5-dimethylthiazol-2-yl)-2,5-diphenyl tetrazolium bromide; NBT: nitro-blue tetrazolium chloride; NCM: nitrocellulose membrane; *O*-glycanase™: Endo-α-*N*-acetylgalactosaminidase; PBS: physiologically buffered saline; PHA: *Phaesolus vulgaris *agglutinin; PNGase A/F: peptide *-N4*-(*N*-acetyl-β-glucosaminyl)-asparagine amidase A/F; RCA_II_: *Ricinus communis *agglutinin; PNA: Peanut agglutinin; SBA: Soybean agglutinin; SD: standard deviation; Ser: serine; SNA: *Sambuca nigra *agglutinin; TBS: tris-buffered saline; TBS-T: tris-buffered saline with Tween 20; Thr: threonine; UEA_I_: *Ulex europaeus *agglutinin; WGA: wheat germ agglutinin.

## Competing interests

The authors declare that they have no competing interests.

## Authors' contributions

SW carried out the experiments and drafted the manuscript. PFB conceived of the study, participated in experimental design and helped draft the manuscript. All authors read and approved the final manuscript.
